# Pyroptosis-Related Gene Signature Predicts Prognosis and Indicates Immune Microenvironment Infiltration in Glioma

**DOI:** 10.3389/fcell.2022.862493

**Published:** 2022-04-25

**Authors:** Yulian Zhang, Chuanpeng Zhang, Yanbo Yang, Guohui Wang, Zai Wang, Jiang Liu, Li Zhang, Yanbing Yu

**Affiliations:** ^1^ Department of Neurosurgery, China-Japan Friendship Hospital, Beijing, China; ^2^ Department of Neurosurgery, Peking University China-Japan Friendship School of Clinical Medicine, Beijing, China; ^3^ Department of Neurosurgery, Graduate School of Peking Union Medical College, Beijing, China; ^4^ Department of Radiotherapy, Tianjin First Center Hospital, Tianjin, China; ^5^ Institute of Clinical Medical Sciences, China-Japan Friendship Hospital, Beijing, China

**Keywords:** pyroptosis, glioma, gene signature, prognosis, tumor microenvironment, immunity

## Abstract

**Objective:** Gliomas are the most common primary tumors in the central nervous system with a bad prognosis. Pyroptosis, an inflammatory form of regulated cell death, plays a vital role in the progression and occurrence of tumors. However, the value of pyroptosis related genes (PRGs) in glioma remains poorly understood. This study aims to construct a PRGs signature risk model and explore the correlation with clinical characteristics, prognosis, tumor microenviroment (TME), and immune checkpoints.

**Methods:** RNA sequencing profiles and the relevant clinical data were obtained from the Chinese Glioma Genome Atlas (CGGA), the Cancer Genome Atlas (TCGA), the Repository of Molecular Brain Neoplasia Data (REMBRANDT), and the Genotype-Tissue Expression Project (GTEx-Brain). Then, the differentially expressed pyroptosis related genes (PRGs) were identified, and the least absolute shrinkage and selection operator (LASSO) and mutiCox regression model was generated using the TCGA-train dataset. Then the expression of mRNA and protein levels of PRGs signature was detected through qPCR and human protein atlas (HPA). Further, the predictive ability of the PRGs-signature, prognostic analysis, and stratification analysis were utilized and validated using TCGA-test, CGGA, and REMBRANDT datasets. Subsequently, we constructed the nomogram by combining the PRGs signature and other key clinical features. Moreover, we used gene set enrichment analysis (GSEA), GO, KEGG, the tumor immune dysfunction and exclusion (TIDE) single-sample GSEA (ssGSEA), and Immunophenoscore (IPS) to determine the relationship between PRGs and TME, immune infiltration, and predict the response of immune therapy in glioma.

**Results:** A four-gene PRGs signature (CASP4, CASP9, GSDMC, IL1A) was identified and stratified patients into low- or high-risk group. Survival analysis, ROC curves, and stratified analysis revealed worse outcomes in the high-risk group than in the low-risk group. Correlation analysis showed that the risk score was correlated with poor disease features. Furthermore, GSEA and immune infiltrating and IPS analysis showed that the PRGs signature could potentially predict the TME, immune infiltration, and immune response in glioma.

**Conclusion:** The newly identified four-gene PRGs signature is effective in diagnosis and could robustly predict the prognosis of glioma, and its impact on the TME and immune cell infiltrations may provide further guidance for immunotherapy.

## Introduction

Gliomas are the most common primary tumors in the central nervous system (CNS), accounting for 81% of intracranial malignancies (Ostrom et al., 2019). Gliomas can be categorized into four grades by according to the 2016 World Health Organization (WHO) classification, among which grade I and II belong to low-grade, grades III and IV indicate high-grade glioma (HGG) (Louis et al., 2016) Glioblastoma (GBM) is the most common type of high-grade glioma (Lara-Velazquez et al., 2017). Despite combing aggressive surgical recession, chemotherapies, and radiotherapy, the prognosis of GBM remains poor, with median overall survival (OS) of 14.6 months (Osuka and Van Meir, 2017). Moreover, the complete resection of GBM is arduous due to the high proliferative rate, heterogeneity of tumor cells, and high infiltrating abilities (Ferguson and Mccutcheon, 2018; Savage, 2018). Recently, more and more molecular markers have been identified that can be used for better diagnosis, treatment and prognostic assessment of glioma patients., including mutations in isocitrate dehydrogenase (IDH), O6-methylguanine-DNA methyltransferase (MGMT) methylation, deletion of the short arm of chromosome 1 and the long arm of chromosome 19 (1p/19q), and various signaling pathways involved in tumor suppression, proliferation, and migration (Wang et al., 2013; Molenaar et al., 2014; Zeng et al., 2015). Over the past decades, numerous therapies have been developed to treat cancers; however, few of them could effectively use in glioma. Despite the blood-brain barrier, unique structure in the CNS (Oberoi et al., 2015), glioma cells adapted various strategies to escape the immune system also play an essential role (Ghouzlani et al., 2021).

Pyroptosis is pro-inflammatory regulated cell death. It is characterized by nuclear condensation, pore formation, cellular swelling, the release of pro-inflammatory cytokines (Shi et al., 2017; Nirmala and Lopus, 2020). Pyroptosis is mediated by gasdermin (GSDM) family proteins. The most studied protein in the GSDM protein family is GSDMD. After being activated by inflammasomes, caspase-1, -4, -5, and -11 can cleave GSDMs into the gasdermin-N and gasdermin-C domains. The gasdermin-N domain (also known as the pore-forming domain) will oligomerize and form pores in the cell membrane (Rathinam and Fitzgerald, 2016). Pyroptosis was first identified in host immune defenses responses. However, supported by a growing number of studies, it gradually became a consensus that pyroptosis also plays an essential role in carcinogenesis and may be a potential anti-tumor target (Munn and Bronte, 2016; Yu et al., 2021; Zhibin Zhang et al., 2021). Pyroptosis could promote anti-tumor inflammatory responses and tumor regression; however, on the contrary, other studies found that pyroptosis may also facilitate the supportive tumor microenvironment. (Hergueta-Redondo et al., 2014; Gao et al., 2018; Wang et al., 2018; Tan et al., 2020; Wang et al., 2021a; Loveless et al., 2021). Thus, the role of pyroptosis remains inconclusive and may differ in different tumors.

Currently, the contribution of pyroptosis related genes (PRGs) as the biomarker for the diagnosis and prognosis of glioma remains unclear. Therefore, we performed a systematic study using multiple expression-level datasets to explore the functional association of PRGs in the prognosis, immune microenvironment, and the response to immunotherapy for glioma patients. This PRGs-based model can predict the prognosis of GBM patients and may further guide the clinical treatment and improve patient survival.

## Methods

### Data Collection

All data used in this study are from public datasets. The RNA sequencing data and corresponding clinical characteristics of glioma patients and RNA-seq data of normal brain tissues were obtained from a combined cohort of The Cancer Genome Atlas (TCGA) and The Genotype-Tissue Expression (GTEx) samples in UCSC Xena project (https://gtexportal.org/home/). The validation datasets were extracted from the Chinese Glioma Genome Atlas (CGGA. http://www.cgga.org.cn/), including mRNAseq_693, mRNAseq_325, and the Repository of Molecular Brain Neoplasia Data (REMBRANDT) dataset. The transcript expression in TCGA-LGG, TCGA-GBM, and GTEx-Brain was recomputed through RNA-Seq by Expectation-Maximization (RSEM) using UCSC TOIL RNA-seq. Gene expression data from CGGA and REMBRANDT were normalized and batched using the R package “limma.”

### Identification of Differentially Expressed Genes

A total of 58 PRGs (listed in Supplementary Table S1) were collected from the MSigDB (https:// www.gsea-msigdb.org/gsea/msigdb) and previous literature (Ju et al., 2021; Lin et al., 2021; Shao et al., 2021; Ye et al., 2021). Differentially expressed genes between gliomas and normal brain tissues was identified using “limma” package with *p*-value < 0.001. The differentially expressed genes (DEGs) are notated as follows: * if *p* < 0.05, ** if *p* < 0.01, and *** if *p* < 0.001. A Protein-protein interaction network and the Gene Ontology (GO) enrichment analysis of candidate genes were performed based on the Metascape online tool (Zhou et al., 2019).

### Consensus Clustering Analysis of Pyroptosis Related Genes

Consensus clustering was applied to explore the connections between the expression of the PRGs and glioma subtypes by the k-means method (Hartigan and Wong, 1979). R packages “ConsensusClusterPlus” and “limma” were used to determine the number of clusters. The correlations between each cluster and clinical characteristics, including OS, were analyzed using the “survival” package. The results were presented by heatmaps and Kaplan-Meier (KM) graphs using R packages “pheatmap,” “survival,” and “survminer.”

### Construction and Validation of a Pyroptosis Related Genes Signature

Sequencing and clinical data from the TCGA were randomly divided into a training set and a testing set according to 1:1 *via* the function “createDataPartition” in the “caret” package. Univariate Cox analysis was first performed to assess the association between the expression of each PRGs and the OS of patients in the TCGA training dataset. *p*-value cutoff was set to 0.05. Then the least absolute shrinkage and selection operator (LASSO) analysis was performed using the R package “glmnet”. Then a prognostic risk formula was constructed by Multivariate Cox regression analysis. The forest plot was performed using the R package “survminer.” The risk scores for each patient were calculated as follows:

$$\mathrm{Risk}\;\mathrm{score}=\sum_{i=1}^{n}\left(\mathrm{Coef}*x_{i}\right)$$

In addition, we accessed the IHC images for each candidate PRGs in glioma and normal tissue samples from the Human Protein Atlas database, and the staining intensity was evaluated according to the standard of HPA database (HPA, https://www.proteinatlas.org/). The Kaplan-Meier curve for each candidate PRGs was generated by using the R package “survminer” to compare the OS between the expression level of these genes.

### Cell Culture

Human cell lines HA1800, U87, T98G, U118, A172, U251, and HMC3 were purchased from ScienCell Research Laboratory, Cell Bank of the Chinese Academy of Sciences and ATCC, and cultured in Dulbecco’s modified Eagle’s medium (DMEM) medium (Invitrogen, Thermo Fisher Scientific, Inc., the United States). All the culture media was supplement by 10% fetal bovine serum (FBS) (Gibco), Penstrep (Gibco), GlutaMAX (Gibco), and MEM non-Essential Amino Acids (MEM-NEAA) (Gibco) following the instruction of the manufacturer. These cells were all cultured in an incubator with 5% CO_2_ at 37°C.

### Validation of the Pyroptosis Related Genes Signature by Quantitative Real-Time PCR

Total RNA from the above cell lines was extracted using Trizol (Beyotime, Shanghai, China). The mRNA concentrations were measured by NanoDrop oneC (Thermo Fisher Scientific, Inc., the United States), next cDNA was synthesized by Hifair® Ⅲ first Strand cDNA Synthesis SuperMix for qPCR(YEASEN Biotech Co., Ltd., China). Quantitative PCR was performed using QuantStudioTM 5 Real-Time PCR System with Hieff ® qPCR SYBR Green Master Mix (YEASEN Biotech Co., Ltd., China). The real-time qPCR was performed as previously described (Zhang et al., 2020a; Zhang et al., 2021d). The corresponding mRNA levels were normalized to GAPDH as an internal control by the 2^−ΔΔCt^ method. The primers used in this study were synthesized by TsingKe biological technology (Beijing, China). The sequence of primers used in this study was listed in Supplementary Table S2.

### Internal and External Validation of the Pyroptosis Related Genes Signature

TCGA datasets, CGGA datasets, and REMBRANDT datasets were used for internal and external validation. The risk scores were calculated for patients with glioma using the formula shown above. Patients were divided into the high- and low-risk groups using the median risk scores as the cutoff value. The time-dependent receiver operating characteristic (ROC) curve was utilized to evaluate the prediction accuracy of PRGs Signature *via* the “timeROC” package. The Kaplan-Meier curve was generated using the R package “survminer” to compare the OS between the high- and low-risk groups. Principal component analysis (PCA) and t-Distributed Stochastic Neighbor Embedding (t-SNE) were performed to explore and visualize the separation the low- and high-risk groups using the “Rtsne” package (Zhou et al., 2019). All these validations were used in the training and validation datasets simultaneously.

### Construction of a Predictive Nomogram

Univariate and multivariate cox regression analyses were performed for the TCGA and CGGA datasets to determine the independent prognostic factors. Next, these clinicopathologic factors were utilized to construct a nomogram to investigate the probability of 1-, 3-, and 5-year OS of patients with glioma using the “rms” package. The C-index, calibration curve, and time-dependent ROC were used to evaluate the consistency and prognostic accuracy of the nomogram.

### Gene Set Enrichment Analyses

The DEGs between the high-risk and low-risk groups were identified with the absolute value of log2FC > 1 and the FDR < 0.05. Gene Ontology (GO) enrichment and Kyoto Encyclopedia of Genes and Genomes (KEGG) pathway analysis were performed via the R package “clusterProfiler,” “org.Hs.eg.db” and “enrichplot” (Yu et al., 2012). Moreover, in TCGA and CGGA datasets, we used the c2.cp.kegg.v7.4.symbols.gmt and c5.go.v7.4.symbols.gmt for gene set enrichment analysis (GSEA) to identify the molecular mechanism that indicated a worse prognosis between the two subgroups (Subramanian et al., 2005).

### Glioma Microenvironment Immune Infiltration Analysis

To investigate the immune microenvironment of glioma, the ESTIMATE algorithm was applied to calculate the immune scores and stromal scores for each sample *via* the R package “estimate” (Yoshihara et al., 2013). Moreover, the scores of infiltrating immune cells and activity of immune-related pathways were quantified through Single sample gene set enrichment analysis (ssGSEA) of the “gsva” package, which contains 29 immune infiltration-related information.

### Immune Response Prediction

Finally, spearman correlation analysis was used to access the relationship between risk scores and the expression levels of common immune checkpoints, including programmed cell death 1 (PD1), programmed cell death-ligand 1 (PD-L1), cytotoxic T-lymphocyte associated protein 4(CTLA-4), Lymphocyte Activating 3 (LAG-3), T-cell immunoglobulin and mucin domain-containing protein 3 (TIM-3), B7-H3(CD276), T-cell immunoglobulin and ITIM domain (TIGIT) (Ghouzlani et al., 2021; Yang et al., 2022) and two newly biomarkers APOBEC3B and TNFSF13 (Zhang et al., 2021a; Zhang et al., 2021c). Then, we investigated the role of PRGs signature in predicting glioma immunotherapeutic response by the tumor immune dysfunction and exclusion (TIDE) algorithm (http://tide.dfci.harvard.edu). A higher TIDE score indicates a worse response to immunotherapy (Jiang et al., 2018). Meanwhile, we use the immunophenoscore (IPS) downloaded from The Cancer Immunome Atlas (TCIA) (https://tcia.at/home) to predict immune checkpoint blockade (ICB) responses in the TCGA-GBM cohort. IPS is a reliable predictor of anti-cytotoxic T-lymphocyte antigen-4 (CTLA-4) and anti-PD-1 antibody responses (Charoentong et al., 2017). Generally, IPS are positively correlated to the ICB response. We recalculated the risk score of patients from the TCGA-GBM cohort according to the PRGs risk model and redivided them into high- and low- relative risk groups based on the median risk score.

### Statistical Analysis

The gene expression differences between the normal brain and glioma tissues were calculated via the Wilcoxon test. The R software (4.0.0) with corresponding packages and GraphPad Prism 8 was used for statistical analyses. *p* < 0.05 was considered statistically significant differences.

## Results

### Characteristics of Patients With Glioma

**FIGURE 1:**
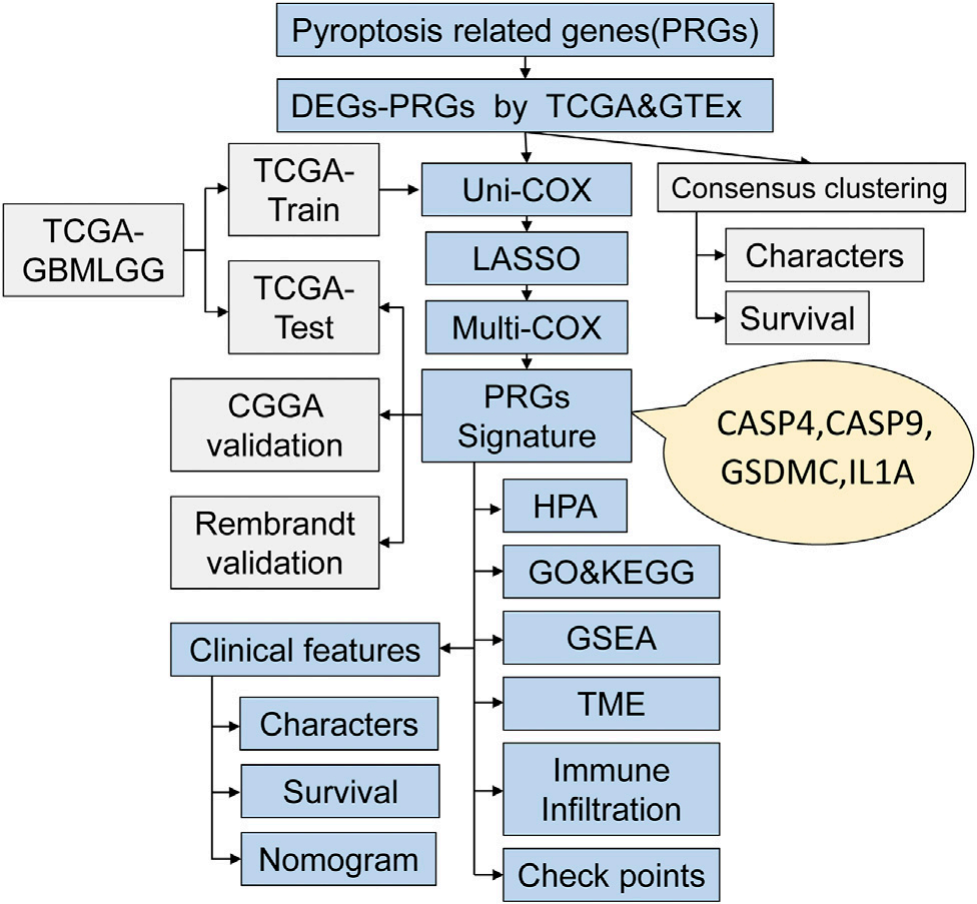
The workflow chart of this study.

The workflow chart of this study is shown in Figure 1. Patients without survival data or histopathological information were excluded. Eventually, 1,137 normal brain and 1,598 glioma tissues were obtained from the GTEx, TCGA-LGG, TCGA-GBM, CGGA, and REMBRANDT datasets. The detailed clinicopathological features of included glioma patients are summarized in Table 1.

**TABLE 1:** Clinicopathological characteristics of glioma patients in TCGA, CGGA, and REMBRANDT datasets.

Characteristics	Train cohort		Validation cohort	
	TCGA-train	TCGA-test	CGGA	Rembrandt
	*N* = 292	*N* = 291	*N* = 715	*N* = 300
Age				
Young	158	161	348	NA
Older	134	130	367	NA
Gender				
Male	176	164	414	146
Female	116	127	301	92
NA	0	0	0	62
Grade				
I	0	0	0	2
II	104	105	186	64
III	124	109	232	57
IV	64	77	297	144
IDH				
Wild	107	111	329	NA
Mutation	185	180	386	NA
1p/19q				
Codel	66	82	147	12
Non-codel	226	209	568	91
NA	0	0	0	197
Status				
Dead	203	210	235	69
Alive	89	81	480	231
MGMT				
Methylated	NA	NA	398	NA
un-methylated	NA	NA	317	NA
PRS				
Primary	NA	NA	458	NA
Recurrent	NA	NA	230	NA
Secondary	NA	NA	27	NA

### Identification of Differentially Expressed Pyroptosis Related Genes Between Normal Brain and Glioma Tissues

**FIGURE 2:**
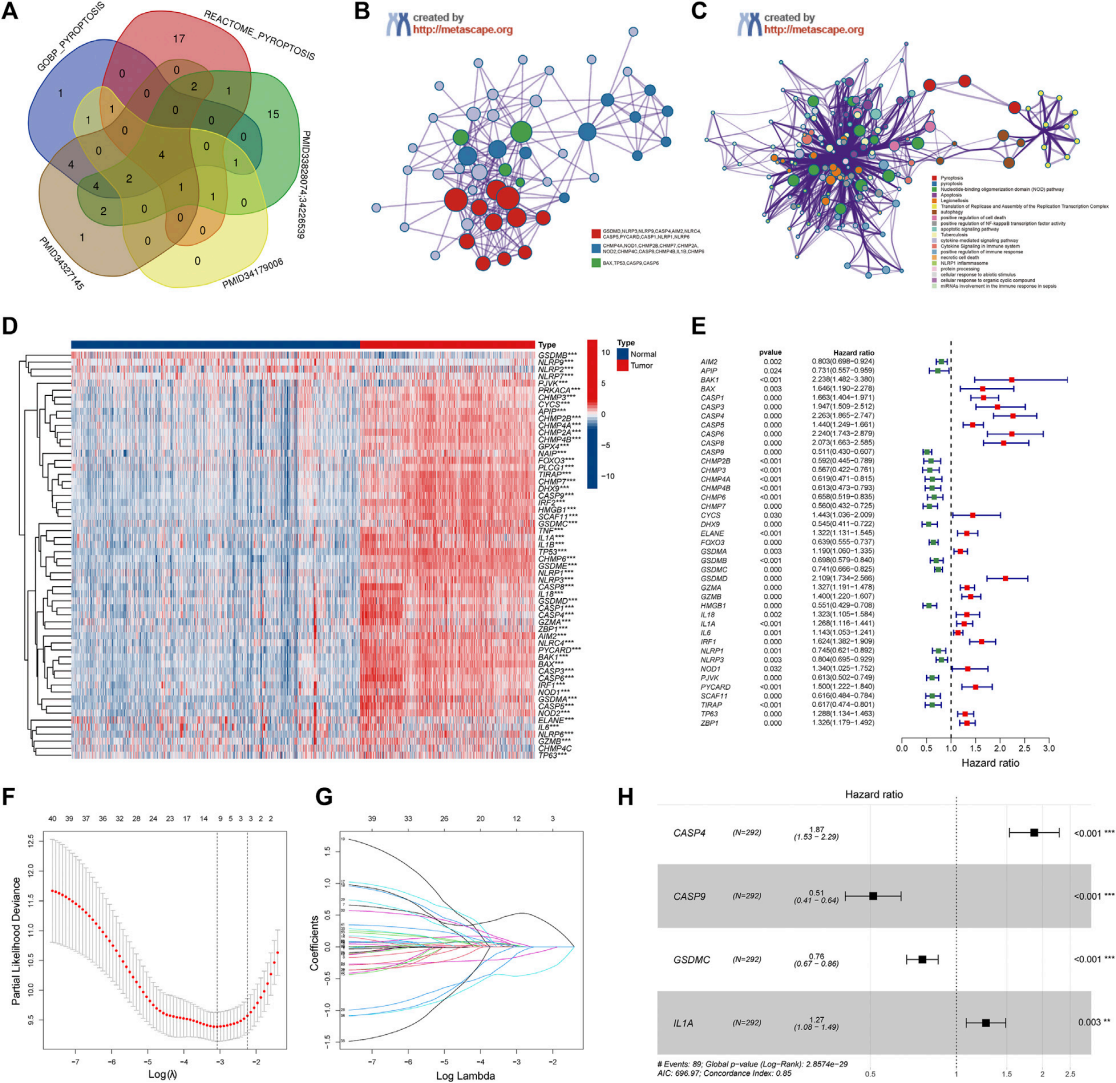
Identification of the Pyroptosis Related genes (PRGs) signature using LASSO-COX regression. **(A)** Venn diagram of the PRGs collected from MSigDB and different literatures. **(B)** Protein-Protein Interaction network was performed by Metascape website. **(C)** Gene Ontology enrichment analysis was performed by Metascape website. **(D)** Heatmap of all PRGs between the normal brain and glioma tissues. **(E)** Forest plot of univariate Cox regression of differential expressed PRGs. **(F)** The log (lambda) sequence plot of PRGs using LASSO regression. **(G)** The LASSO coefficient profiles of PRGs in TCGA-train dataset. **(H)** Forest plot of four signature genes of PRGs. LASSO, least absolute shrinkage, and selection operator; TCGA, The Cancer Genome Atlas. **p < 0.01, ***p < 0.001.

A total of 58 PRGs were collected for our analyses (Figure 2A). To further explore the interactions of these differentially expressed PRGs, we used the Metascape website to construct a PPI network. GO enrichment analysis was also performed to investigate the enriched pathways. As expected, these PRGs were involved in pyroptosis pathways (Figures 2B,C). Wilcoxon test was performed to compare the expression levels of 58 PRGs between the normal brain and glioma tissues. The results show that 57 out of 58 were significantly differentially expressed (*p*-value < 0.001) (Figure 2D). Three genes (NLRP2, NLRP9, and GSDMB) were downregulated, while the remaining 54 genes were upregulated in the glioma group. The RNA levels of these genes were displayed in heatmaps.

### Identification of Glioma Clusters Using Consensus Clustering

To investigate the correlation between the expression of PRGs and clinicopathological characteristics, we performed consensus cluster analysis with 583 glioma patients from the TCGA datasets (Supplementary Figure S1A). The Cumulative Distribution Function (CDF) and delta area under CDF change was utilized to determine a suitable clustering variable (k) for the optimum stability of the sample distribution (Supplementary Figures S1B,C). The patients were divided into two distinct clusters, and the OS of cluster 1 was significantly better than cluster 2 (*p* < 0.001) (Supplementary Figure S1D). Besides, as shown in the heatmap (Supplementary Figure S1E), except for the gender, there were significantly differences in clinicopathologic features between the two clusters, including the age, grade, IDH status, and 1p19q codeletion (*p* < 0.001).

### Construction of a Prognostic Model Using the Training Dataset

Patients in the TCGA cohort were randomly divided into a training group (*n* = 292) and a testing group (*n* = 291). Univariate Cox regression analysis was performed among these 57 PRGs in the training datasets to screen prognostic genes, and 41 genes remained for further analysis (*p* < 0.05). Among them, 19 were protective genes (hazard ratio <1), and 22 genes were associated with increased risk (hazard ratio >1) (Figure 2E). Subsequently, the LASSO method was used to narrow down the candidate genes according to the minimum penalty parameter (λ) (Figures 2F,G). Finally, combined with Multivariate Cox regression analyses, four PRGs (CASP4, CASP9, GSDMC, IL1A) were identified as prognostic biomarkers for glioma patients. The PRGs signature risk model was formulated as: Risk score = (0.629*CASP4 exp.) + (−0.670* CASP9 exp.) + (−0.274 * GSDMC exp.) + (0.237* IL1A exp.) (Figure 2H).

### The Relationship Between the Expression Status of the Pyroptosis Related Genes Signature and the Tumor Grade and Prognosis

**FIGURE 3:**
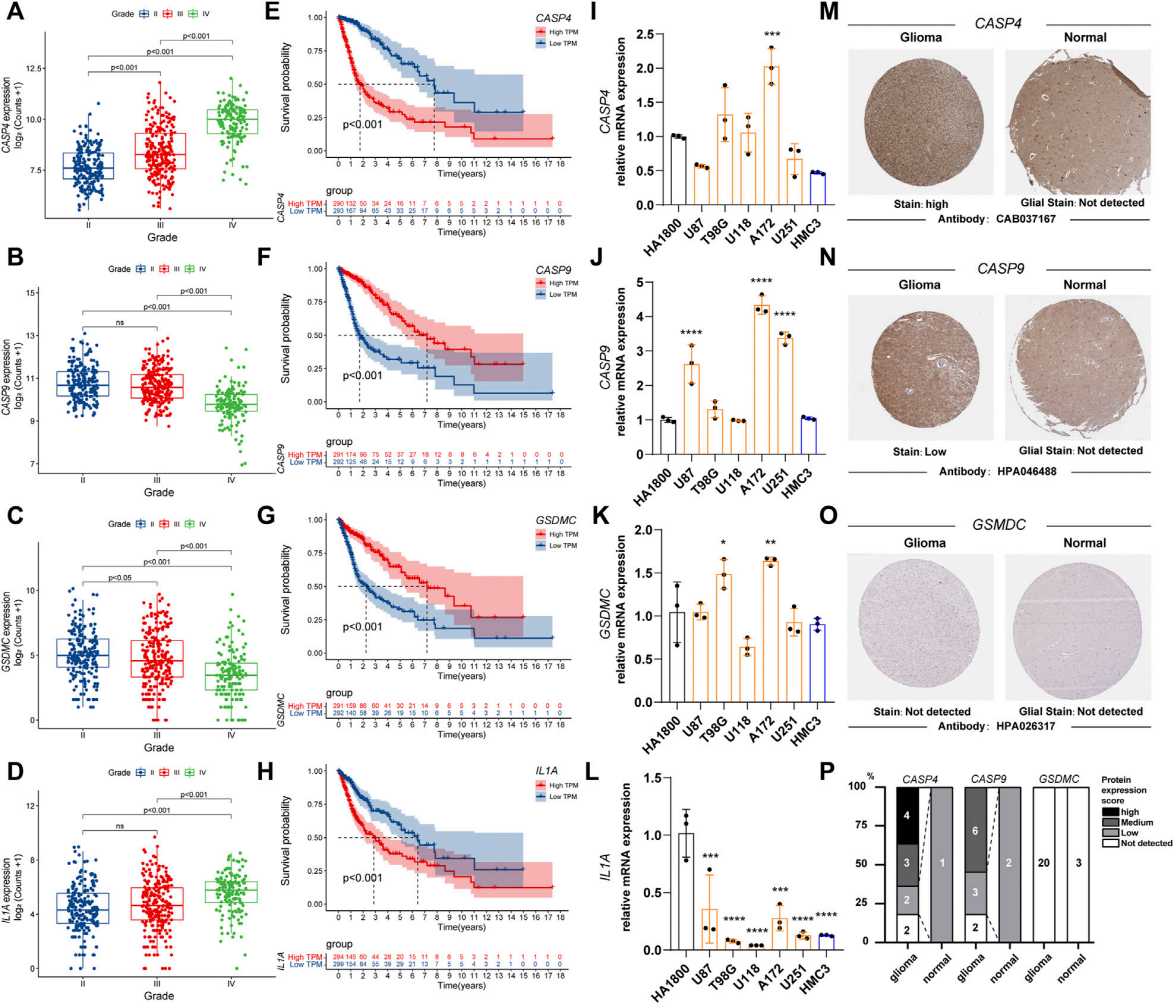
The relationship between the expression status of the PRGs signature and the tumor grade and prognosis. **(A–D)** Box plot of PRGs signature expression with tumor grade. **(E–H)** Overall survival of different PRGs signature expression in glioma. **(I–L)** Relative mRNA expression of PRGs signature in different cell lines by qPCR. (*p < 0.05, **p < 0.01, ***p < 0.001. ****p < 0.0001, Not labeled: no statistical significance compared to HA 1800). **(M–O)** Representative protein expression images of PRGs signature by the Human Protein Atlas (HPA). **(P)** Statistical column stacking diagram of PRGs signature protein expression in HPA. PRGs, Pyroptosis Related genes. qPCR, quantitative Polymerase Chain Reaction. PRGs, Pyroptosis Related genes.

We observed the relationship between the expression status of the four genes and the tumor grade and prognosis. The results suggest that high expression of CASP4 and IL1A is associated with higher WHO classification (Figures 3A,D), in contrast to CASP9 and GSDMC (Figures 3B,C). For prognosis, high expression of CASP4 and IL1A was associated with worse prognosis (Figures 3E,H), while CASP4 and GSDMC were not (Figures 3F,G). Further, we observed the mRNA expression of the four genes in different cell lines by qPCR. Compared with HA 1800 and HMC3 cell lines, the expression levels of CASP4, CASP9 and GSDMC showed an overall upward trend, and the IL1A level showed an overall downward trend in glioma cell lines (Figures 3I–L). In addition, we explored the proteins express by CASP4, CASP9, and GSDMC in glioma patients through the Human Protein Atlas database, and CASP4, CASP9 exhibited higher staining intensity in glioma than normal brain tissues (Figure 3M–O). The statistical histogram was shown in Figure 3P.

### Internal and External Validation of the Pyroptosis Related Genes Signature

**FIGURE 4:**
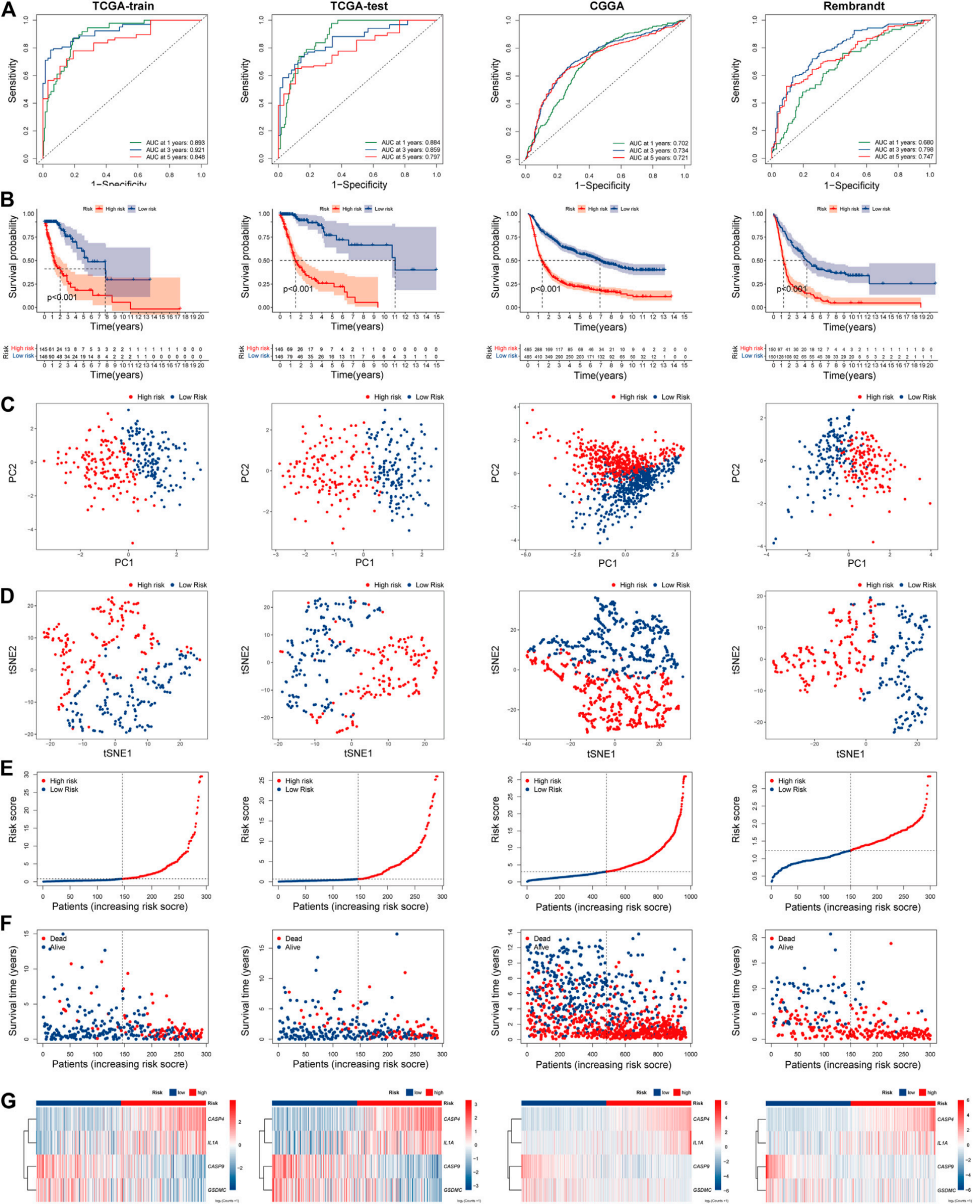
Prognostic risk model analysis of PRGs signature in TCGA-train, TCGA-test, CGGA, and Rembrandt datasets. **(A)** The time-dependent ROC analyses of the PRGs risk score in TCGA-train, TCGA-test, CGGA, and Rembrandt datasets. **(B)** Survival curve of the PRGs signature risk model in above datasets. **(C)** PCA analysis of the PRGs signature in above datasets. **(D)** t-SNE analysis of the PRGs signature in above datasets. **(E)** The distribution and median value of the PRGs signature risk scores in above datasets. **(F)** The distributions of survival status, survival time, and PRGs risk score in above datasets. **(G)** Heatmap of PRGs signature expression in high- and low- risk groups in above datasets. REMBRANDT, Repository of Molecular Brain Neoplasia Data. PRGs, Pyroptosis Related genes. ROC, receiver operating characteristic; TCGA, The Cancer Genome Atlas. CGGA, Chinese Glioma Genome Atlas. PCA, Principal components analysis. t-SNE, t-Distributed Stochastic Neighbor Embedding.

The risk scores of each patient were calculated based on the signature constructed. Then, glioma patients were stratified into the high- and low-risk groups by median risk score. In the TCGA train group, time-dependent ROC curves were used to evaluate the sensitivity and specificity of the prognostic model, and the area under the ROC curve (AUC) for 1-, 3, - and 5-year OS was 0.893, 0.921, and 0.848, respectively (Figure 4A left). Kaplan-Meier survival curve showed that the OS of patients in the high-risk group was significantly shorter than the high-risk group (*p* < 0.001; Figure 4B left). PCA and t-SEN analysis revealed a satisfactory separation between different risk groups (Figures 4C,D left). The distribution of patients was presented (Figures 4E,F left). Patients in the low-risk group had a lower mortality rate and longer lifespan. The expression of these four PRGs in different groups was shown in the heatmap (Figure 4G left). CASP4 and IL1A were upregulated in the high-risk group, while CASP9 and GSDMC were downregulated. Subsequently, similar analyses were performed using TCGA-test, CGGA and Rembrandt databases (Figures 4A–G right).

### Relationship Between the Risk Group of Pyroptosis Related Genes Signature and Clinicopathological Characteristics of Gliomas

**FIGURE 5:**
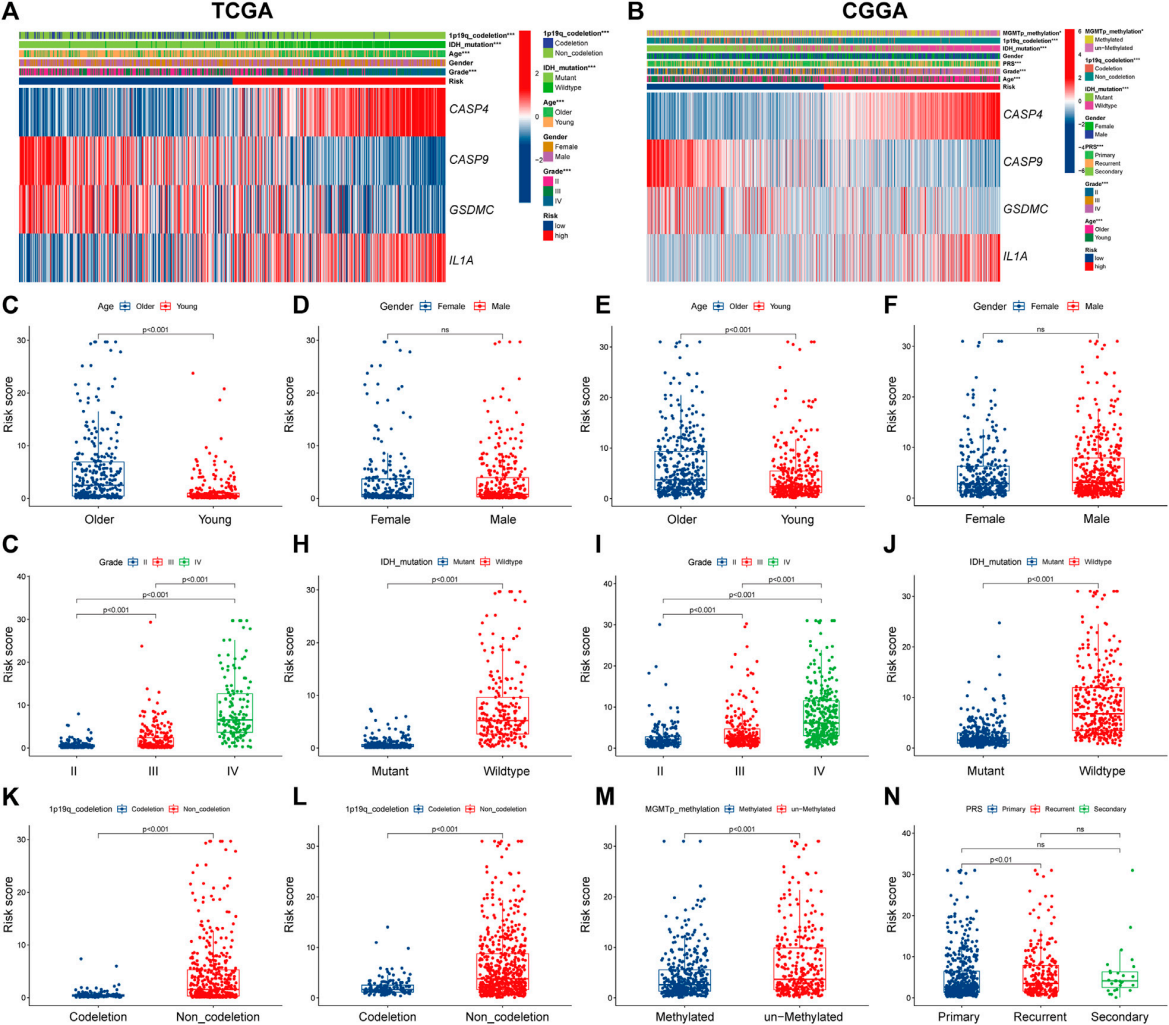
The correlation between PRGs risk score and clinicopathologic characteristics. **(A,B)** Heatmap of correlation between PRGs risk groups with age, gender, tumor grade, IDH status, 1p19q codeletion, and PRGs signature gene expression in TCGA dataset **(A)**, and age, gender, tumor grade, IDH status, 1p19q codeletion, MGMT promoter methylation status, PRS type, and PRGs signature gene expression in CGGA dataset **(B)**. **(C–N)** Box and scatter plot of relationship between risk score and age **(C)**, gender **(D)**, tumor grade **(G)**, IDH status **(H)**, and 1p19q codeletion **(K)** in TCGA dataset; and age **(E)**, gender **(F)**, tumor grade **(I)**, IDH status **(J)**, 1p19q codeletion **(L)**, MGMT promoter methylation status **(M)**, and PRS type **(N)** in CGGA dataset, respectively. PRGs, Pyroptosis Related genes. TCGA, The Cancer Genome Atlas. CGGA, Chinese Glioma Genome Atlas. IDH, isocitrate dehydrogenase. MGMT, O6-methylguanine-DNA methyltransferase. PRS, tumor type of primary recurrent or secondary.

Next, we explored the correlation between PRGs signature and clinicopathological features of gliomas in TCGA and CGGA datasets. Significant differences were observed between the two risk subgroups regards to WHO grade (II, III, IV), age (<47, ≥47), IDH status (mutation, wild), and 1p19q codeletion in the TCGA cohort (Figure 5A). Similar analyses were performed using CGGA (Figure 5B). Subsequently, we compared the risk score across patients with different clinicopathological characteristics. For the TCGA cohort, glioma patients with the clinicopathological features of age >47 years, higher grade, IDH wild type, and 1p19q non-codeletion had significantly higher risk score levels (Figures 5C,G,H,K). In addition, no significant difference was observed between groups stratified by gender (Figure 5D). Similar results were observed in the CGGA cohort (Figures 5E,F,I,J,L,N); besides, MGMT promoter unmethylated and recurrent subgroups also had significantly higher risk scores (Figure 5M).

### Nomogram

**FIGURE 6:**
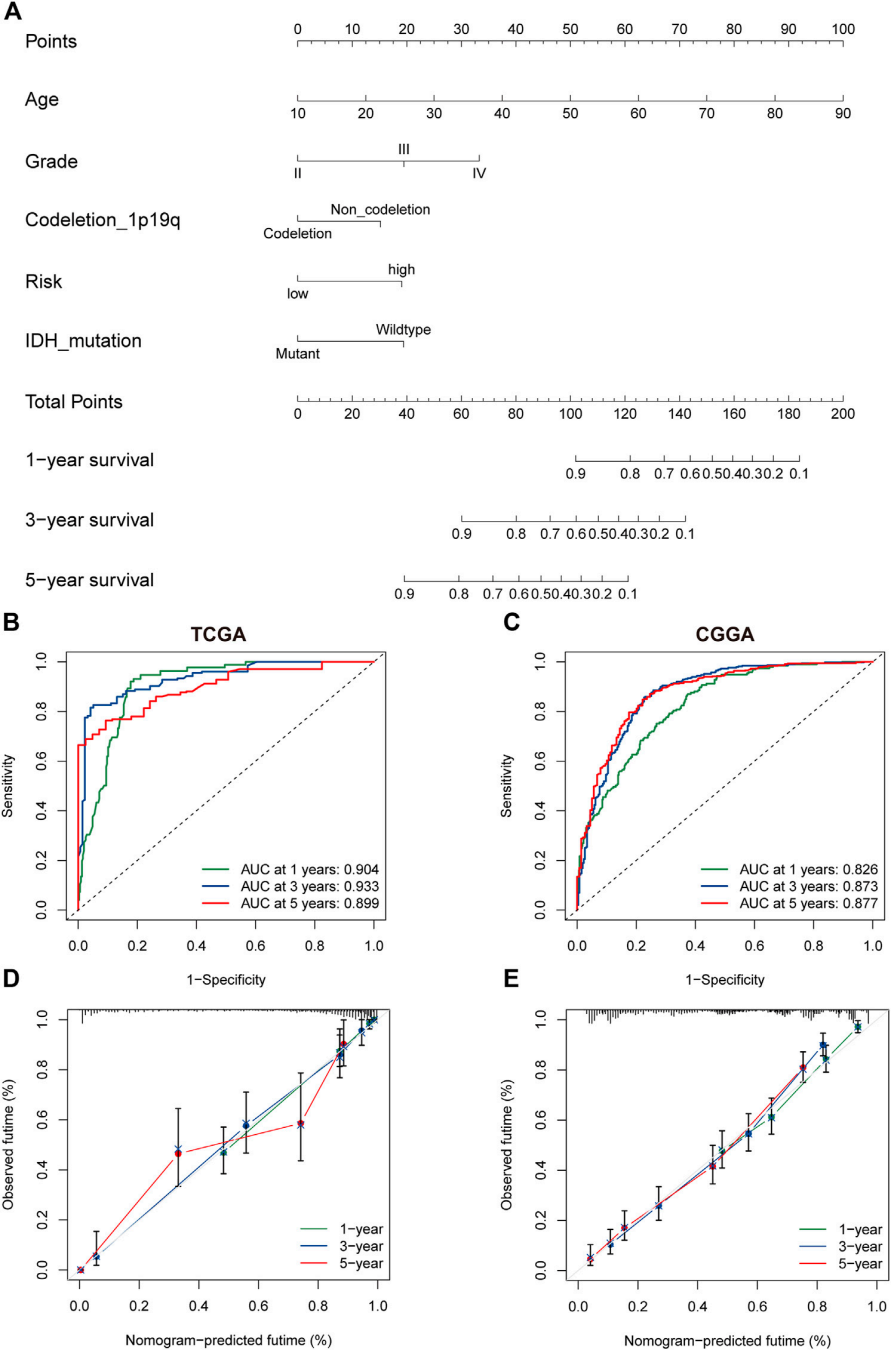
The nomogram of the PRG signature predicts survival in glioma patients. **(A)** The nomogram with PRGs signature risk group for the prediction of OS of glioma patients was constructed based on the TGGA dataset. BC. The time-dependent ROC analyses of the PRGs nomogram in TCGA **(B)**, and CGGA **(C)**. **(D,E)** The calibration plots for predicting 1-, 3-, and 5-year survival using TCGA **(D)**, and CGGA **(E)**. PRGs, Pyroptosis Related genes. TCGA, The Cancer Genome Atlas. CGGA, Chinese Glioma Genome Atlas.

The univariate and multivariate cox regression analyses were performed to identify PRGs signatures as independent OS-related predictors. For the TCGA cohort, Univariate Cox regression analysis demonstrated that the risk scores were associated with the OS of glioma patients (HR = 6.793, 95%CI = 4.600-10.032, *p* < 0.001). Multivariate Cox regression analysis revealed that the risk-score was an independent prognostic factor (HR = 2.048, 95%CI = 1.192 −3.517, *p* < 0.001). The results were similar in the CGGA cohort (Supplementary Figure S2A–D). Subsequently, a nomogram based on clinical characteristics, including age, WHO grade, IDH status, 1p19q codeletion, and risk score in the TCGA cohort was established (Figure 6A). The area under the ROC curve (AUC) for 1-, 3-, and 5-year OS was 0.904, 0.933, and 0.899, respectively (Figure 6B). In addition, the internal assessment showed a consistency index (C-index) of 0.866, and the calibration curve showed a satisfactory match between the actual and nomogram-predicted 1-year, 3-year, and 5-year OS probabilities (Figure 6C). The same analysis was performed in the CGGA cohort as an external evaluation. The accuracy of the nomogram in predicting 1-, 3-, and 5-year OS was 0.826, 0.873, and 0.877, respectively (Figure 6D). Additionally, the C-index was 0.744, and calibration curves show a satisfactory match between the actual and nomogram-predicted probabilities of 1-year, 3-year, and 5-year OS (Figure 6E).

### Functional Enrichment Analyses

**FIGURE 7:**
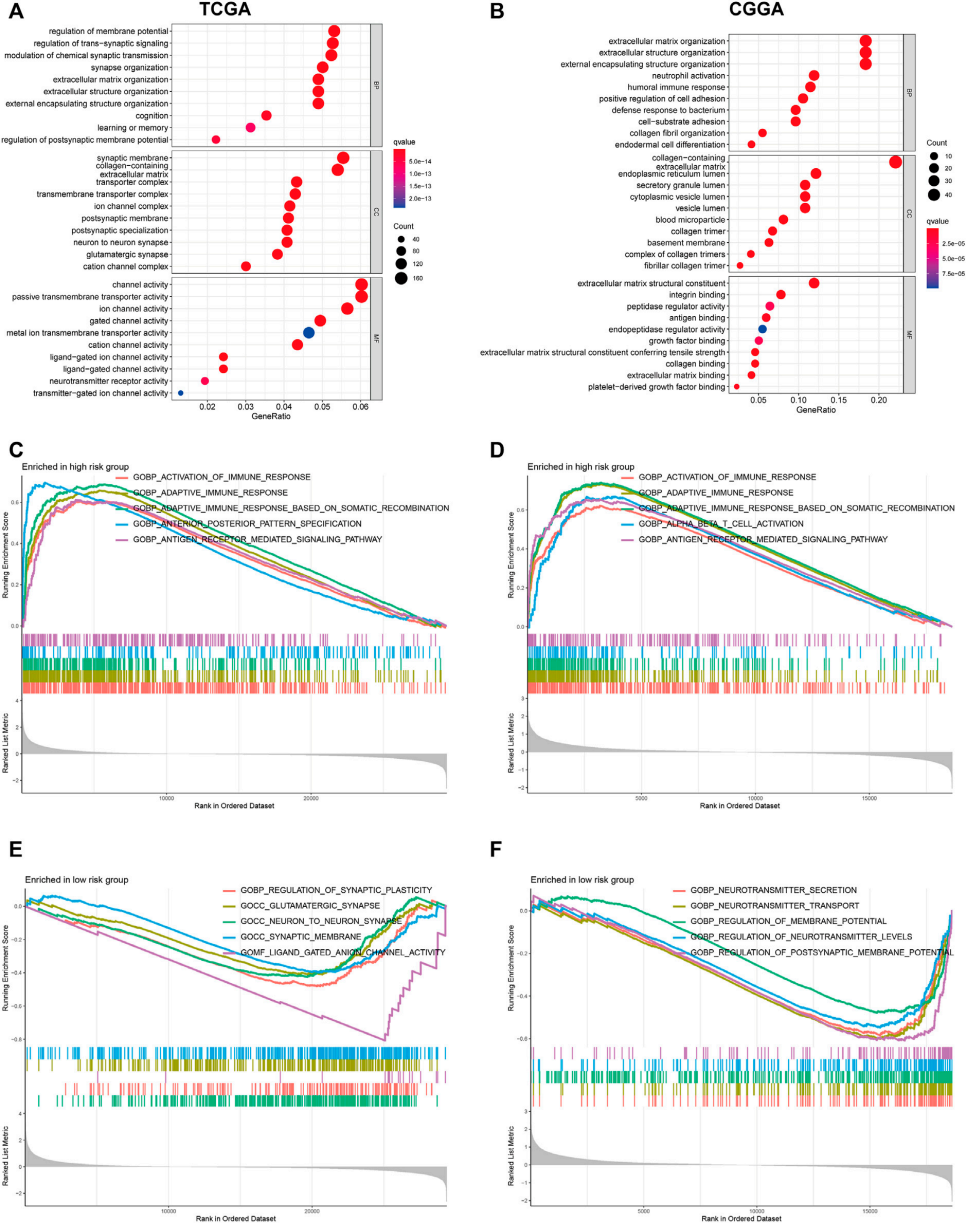
GO functional enrichment analyses between different PRGs risk groups. **(A,B)** Bubble graph of GO enrichment analysis between high- and low- PRGs risk group using TCGA **(A)** and CGGA **(B)**. **(C–F)** GSEA GO analyses of different PRGs risk group in TCGA or CGGA datasets. **(C)**: high risk, TCGA; **(D)**: high risk, CGGA; **(E)**: low risk, TCGA; **(F)**: low risk, CGGA). GO, Gene Ontology. PRGs, Pyroptosis Related genes. TCGA, The Cancer Genome Atlas. CGGA, Chinese Glioma Genome Atlas. GSEA, gene set enrichment analysis.

To clarify the potentially functional mechanism of the PRGs and prognosis of patients with glioma, GO, and KEGG enrichment analyses were conducted to characterize the biological functions of DEGs between low- and high-risk groups. GO analyses in the TCGA cohort showed gene enrichment in trans-synaptic signaling regulation (Figure 7A), and in CGGA datasets revealed significant enrichment of immune-related biological processes, including neutrophil activation, humoral immune response, and defense response to bacterium (Figure 7B). Similarly, KEGG pathway analysis showed an enrichment of immune-related pathways in both cohorts (Supplementary Figures S3A,B). For instance, cytokine-cytokine receptor interaction and antigen processing and presentation pathway. To further verify these results, GSEA was performed in both cohorts. In the c5.go dataset, both cohorts showed significant enrichment of immune-related biological processes in the high-risk group, for instance, activation of the immune response, adaptive immune response, and adaptive immune response (Figures 7C–F). In the c2.cp.kegg dataset, the high-risk group was also significantly associated with immune-related pathways, such as cytokine-cytokine receptor interaction, JAK-STAT signaling pathway, and chemokine signaling pathway (Supplementary Figure S3C–F).

### Immune Cell Infiltration in the Tumor Microenvironment

**FIGURE 8:**
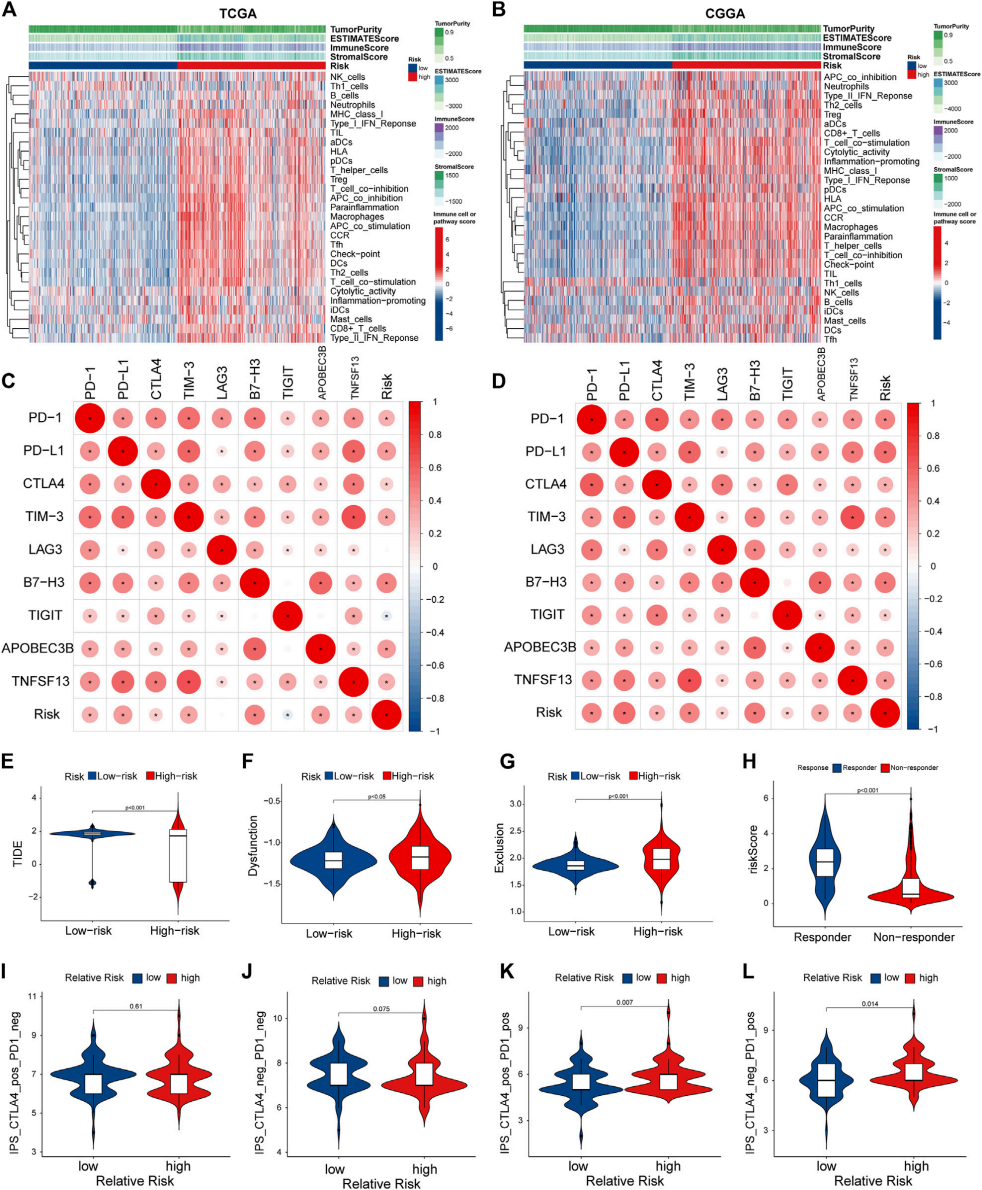
Tumor microenvironment and immunocorrelation analysis of PRGs signature risk group in glioma. **(A,B)** Heatmap of correlation between different risk group and different immune infiltration pattern using ssGSEA in TCGA **(A)** and CGGA **(B)**. **(C,D)** Correlation analysis between PRGs risk score and expression levels of immune checkpoints (PD1, PD-L1, CTLA-4, LAG-3, TIM -3, B7-H3, TIGIT, APOBEC3B, and TNFSF13) in TCGA **(C)** and CGGA **(D)**. **(E–H)** Correlation analysis between TIDE evaluation in TCGA(LGG-GBM) datasets. TIDE score **(E)**, dysfunction score **(F)**, Exclusion **(G)** and Responder **(H)**. **(I–L)** Correlation analysis between immunophenoscore of anti-cytotoxic T-lymphocyte antigen-4 (CTLA-4) and anti-PD-1 blocker and Relative risk of TCGA-GBM datasets. TIDE, Tumor Immune Dysfunction and Exclusion. PRGs, Pyroptosis Related genes. TCGA, The Cancer Genome Atlas. CGGA, Chinese Glioma Genome Atlas. ssGSEA: single sample gene set enrichment analysis.

Considering that the enrichment analysis identified multiple immune-related pathways, we further investigated the correlation between PRGs signature and immune cell infiltration in the glioma microenvironment. In both TCGA and CGGA cohorts, the high-risk group showed significantly lower tumor purity and higher immune and stromal scores than the low-risk group (Figures 8A,B). Next, we performed the ssGSEA analysis to determine the correlation between the PRGs signature and immune cell infiltration in the tumor microenvironment. In the TCGA cohort, except for the neutrophils, natural killer (NK) cells, and Th1 cells, the other 13 immune cells showed significantly higher levels of infiltration in the high-risk group (Supplementary Figure S4A). In comparison, all the 16 types of immune cells showed a higher abundance in the CGGA cohort (Supplementary Figure S4B). Moreover, the activity of 13 immune-related pathways was higher in the high-risk subgroup of both TCGA and CGGA cohorts (Supplementary Figures S4C,D). We further explored the correlation between the risk score and immune checkpoints and some newly biomarkers (PD1, PD-L1, CTLA-4, LAG-3, TIM -3, B7-H3, TIGIT, APOBEC3B, and TNFSF13). The Spearman analysis showed that the risk score was significantly correlated with all nine immunotherapy checkpoints. In the TCGA cohort, the risk score was negatively correlated with TIGIT, and the other six genes were positively correlated (Figure 8C). Meanwhile, in the CGGA cohort, all of them were positively correlated with the risk score (Figure 8D). To better elucidate the value of PRGs signature in predicting immunotherapy response, we analyzed TIDE scores in gliomas patients of TCGA. The results showed that the TIDE and dysfunction scores were lower in the high-risk group (Figures 8E,F), and exclusion scores showed the opposite trend (Figure 8G). Moreover, the responder group was positively associated with risk scores, indicating that patients in the high-risk group might have a better response to immunotherapy (Figure 8H). The immunological score could predict the anti-CTLA-4 and anti-PD-1 antibody response, which can identify determinants of tumor immunogenicity (Charoentong et al., 2017). Subsequently, we investigated this correlation of immunophenoscore in the TCGA- GBM cohort and found that risk groups in IPS-CTLA4 and IPS had no significant difference in immunophenoscore (Figures 8I,J). Whileas, IPS- PD1 and IPS-PD1-CTLA4 blocker scores were higher in the high-risk group suggesting better immunotherapeutic benefits (Figures 8K,L).

## Discussion

Extensive research on the mechanism of the pyroptosis pathway has led to a renewed interest in accessing the effect of pyroptosis for cancer treatment owing to its pro-inflammatory effects (Hou et al., 2021). Emerging studies have revealed that pyroptosis-related gene signatures were associated with anti-tumor immunity and could be used to predict patient prognosis in a variety of cancer types. (Ju et al., 2021; Lin et al., 2021; Shao et al., 2021; Ye et al., 2021). However, no previous study has been conducted to demonstrate the predictive value of pyroptosis-related genes in glioma. Considering the emerging role of pyroptosis in carcinogenesis (Loveless et al., 2021) and the poor prognosis of glioma, we built a new tool based on pyroptosis signatures to establish a predictive model for glioma.

In this study, we compared the expression level of 58 PRGs between glioma and normal brain tissue samples, and we found that 57 of 58 genes were differentially expressed. To investigate the prognostic value of the expression of PRGs in glioma, we performed consensus clustering, which showed that patients could be divided into two clusters based on different clinical characteristics. Then we selected 4 PRGs (CASP4, CASP9, GSDMC, IL1A) based on a signature predictive model via Univariate Cox proportional hazards regression, LASSO regression, and multivariate Cox regression. For internal and external validation datasets, the results suggest the risk model could accurately predict survival outcomes of patients with glioma. Combined with other Clinicopathological features (age, grade, IDH, 1p19q), we built a nomogram with the improved capacity to predict the overall survival rate. Functional enrichment analyses indicated the differences in immune-related biological processes between high- and low-risk groups. We further investigated the difference in tumor immune microenvironment between the two groups. The results showed that the high-risk group had lower tumor purity and higher immune and stromal scores. Immune cell infiltration analysis revealed that risk score was positively correlated with the level of immune cell and immune-related pathways. Moreover, PRGs signatures may related to the benefits of immunotherapy.

Two fundamental and several alternative pathways are associated with pyroptosis, in which, caspase-1activates the canonical inflammasome pathway, non-canonical inflammasome pathway *via* caspase-4/5 (or mouse caspase-11), and in the alternative pathways, the most reported one is caspase-3 (Fang et al., 2020; Loveless et al., 2021). GSDM family proteins consist of an N-terminal pore-forming domain, a C-terminal regulatory domain, and a linker region that inhibit the N-terminal domain’s lethal activity (Kuang et al., 2017). Caspases or granzymes can cleave the linker site, resulting in the N-terminal domain fragment translocating into cell membranes. Thus, the N-terminal domain triggers the oligomerization and forms β -barrel transmembrane pores, leading to cytokines released following cell lysis, such as interleukin-1 β (IL-1 β) and interleukin-18 (IL-18) (Guey et al., 2014; Ding et al., 2016). The available studies suggest that 4 PRGs we found (CASP4, CASP9, GSDMC, and IL1A) may be involved in different pathways of pyroptosis.

In the nonclassical pyroptosis pathway, human CASP-4, -5 and murine orthologues CASP-11 can be directly activated by cytosolic lipopolysaccharide (LPS) from Gram-negative bacteria or host-derived oxidized phospholipids. Activated caspase-4/5/11 then cleave GSDMD to generate biologically active GSDMD-NT, contributing to pyroptotic cell death (Shi et al., 2014; He et al., 2015; Kayagaki et al., 2015; Gao et al., 2018; Wang et al., 2018; Ahmed et al., 2019; Qiao et al., 2019). It is reported that GSDMB could bind to the CARD of caspase-4, trigger its oligomerization, and increase its enzymatic activity, thereby promoting the cleavage of GSDMD and inducing non-canonical pyroptosis (Chen et al., 2019). Our study showed that CASP4 was negatively correlated with OS and positively correlated with the WHO grade of glioma patients, suggesting that CASP4 may act as an oncogene in glioma.

CASP9 is a classical initiator of intrinsic apoptosis (Li et al., 1997). Recent studies showed that GSDME could be activated by caspase-3/-9 in lung cancer cells and melanoma (Zhou et al., 2018; Zhang et al., 2019). Mitochondrial dysfunction could trigger caspase-9 activation and subsequently cleaves and activates caspase-3. Active caspase-3 then cleaves GSDME to GSDME-NT, thus leading to pore-formation on the cell membrane, which is the hallmark for pyroptosis (Wang et al., 2021b). When GSDME is defective, cells are more prone to apoptosis. However, in cells with high GSDME expression, GSDME-mediated pyroptosis may precede apoptosis. (Wang et al., 2017).

IL-1α, a pre-stored cytokine of the IL-1family, is a canonical immune alarmin passively released during cell lysis (Monteleone et al., 2015). Our study showed that IL1A is negatively correlated with OS and positively correlated with WHO classification of glioma patients. Previous studies have demonstrated pro–IL-1α is bound to the intracellular receptor IL-1R2, which was usually processed by calpain during necrosis, and can also be cleaved to its mature form by caspase-5 and -11 in pyroptosis (Zheng et al., 2013; Wiggins et al., 2019). Batista et al. revealed that IL-1α is expressed in microglia and *ex vivo* IL-1α release is dependent on GSDMD, which promotes protective immunity in brain inflammation and parasite infection. (Batista et al., 2020). Under the inhibition of caspase-1, the activation of NLRP3 inflammasome induces incomplete pyroptosis and is accompanied by IL-1α release (Chan and Schroder, 2020).

Early studies found GSDMC was highly expressed in metastatic melanoma cells (Watabe et al., 2001; Miguchi et al., 2016; Wei et al., 2020). However, GSDMC was downregulated in GC and esophageal cancer cells and inhibited cell growth (Saeki et al., 2009). Recently, Hou et al. reported that GSDMC could be cleaved and activated in breast cancer cells by caspase 8, converting TNF-α-induced apoptosis to pyroptosis, leading to tumor necrosis (Hou et al., 2020). Our study showed that GSDMC is positively correlated with OS and negatively correlated with WHO classification of glioma patients, suggesting that GSDMC may act as a tumor suppressor in glioma. Considering the complex role of GSDMC in different tumors, further studies should be conducted to validate the functional mechanisms of GSDMC in glioma cell and animal models.

The immunosuppressive microenvironment and the resistance to apoptosis are significant factors contributing to the therapeutic dilemma of the tumor (Loveless et al., 2021). Cancer cell pyroptosis may promote immune cell activation and infiltration, eliciting a robust inflammatory response, thus leading to immunogenic cell death (ICD) (Zhang et al., 2020b). However, given its inflammatory feature, pyroptosis possibly induces an immunosuppressive microenvironment in some conditions, such as pathogen infection or hypoxia (Hou et al., 2020; Hou et al., 2021). The effect of pyroptosis may be determined by the complex interactions between tumor cells and the surrounding microenvironment. Previous studies have shown that therapeutic inhibition of IDO, CTLA-4, or PD-L1 in mouse glioma models significantly reduces the number of tumor-infiltrating Treg cells and substantially improves long-term survival. (Wainwright et al., 2014). Immune checkpoint blockade appears to be a promising strategy in the immunotherapy of glioma. The application of various immunotherapeutic approaches, especially combination strategies, has been shown to be efficacious in glioma (Zhang et al., 2021b; Yang et al., 2022). Currently, nanoparticles loaded with pyroptosis inducers have been shown to be effective for treating tumors in both *in vivo* and *in vitro* models and activating immunity in breast cancer (Wang et al., 2020). In our study, DEGs between different risk subgroups were enriched in many biological processes and pathways related to immune responses. Besides, we found that the high-risk group exhibited lower tumor purity and higher immune and stromal scores, a higher abundance of immunosuppressive cells such as Tregs, and higher expression levels of immune checkpoints. These results suggested that the PRGs were correlated with the immune landscape of the glioma microenvironment. Our findings may provide new ideas and targets for the immunotherapy of glioma.

Undoubtedly, some limitations must be addressed in the present study. Firstly, our current results are obtained merely in public databases, and it is necessary to validate our results *in vitro* and *in vivo*. Secondly, although bulk genomic and transcriptome analyses provide valuable insights into this study, the information obtained at the bulk level is averaged over many cells number, often masking specific subpopulations or cell states. Therefore, the relationship between glioma pyroptosis and the local immune microenvironment may require an in-depth study by techniques such as single-cell sequencing. Besides, the available samples for qPCR were not sufficient. In further work, more tissue samples is needed to reach a robust result.

## Conclusion

This study built a prognostic model based on PRGs signature. This model exhibited high accuracy in predicting survival outcomes of glioma patients and was validated by external data sources. In addition, the PRGs signature was correlated with immune infiltration of the glioma microenvironment and was indicative of different efficacy of immunotherapy to a certain extent. These findings will offer some valuable insights for subsequent studies and clinical practice.

## Data Availability

The datasets presented in this study can be found in online repositories. The names of the repository/repositories and accession number(s) can be found in the article/Supplementary Material.
