# Supplementary figures and images for: Pyroptosis-Related Gene Signature Predicts Prognosis and Indicates Immune Microenvironment Infiltration in Glioma
Source: Front Cell Dev Biol. 2022 Apr 25;10:862493. doi: 10.3389/fcell.2022.862493 (PMC9081442; doi:10.3389/fcell.2022.862493)

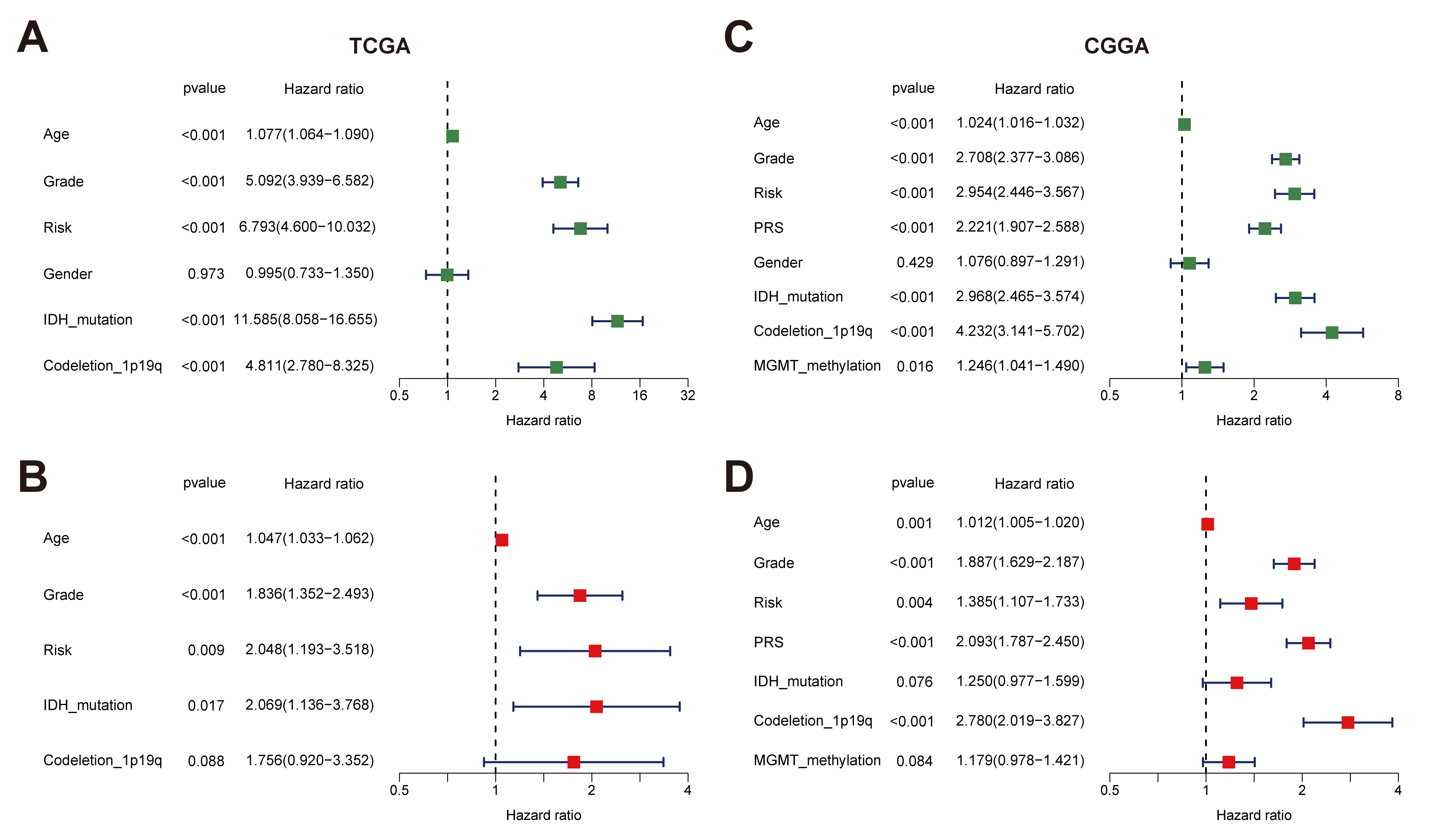

Supplement: Supplementary file 2 [file Image3.TIF]

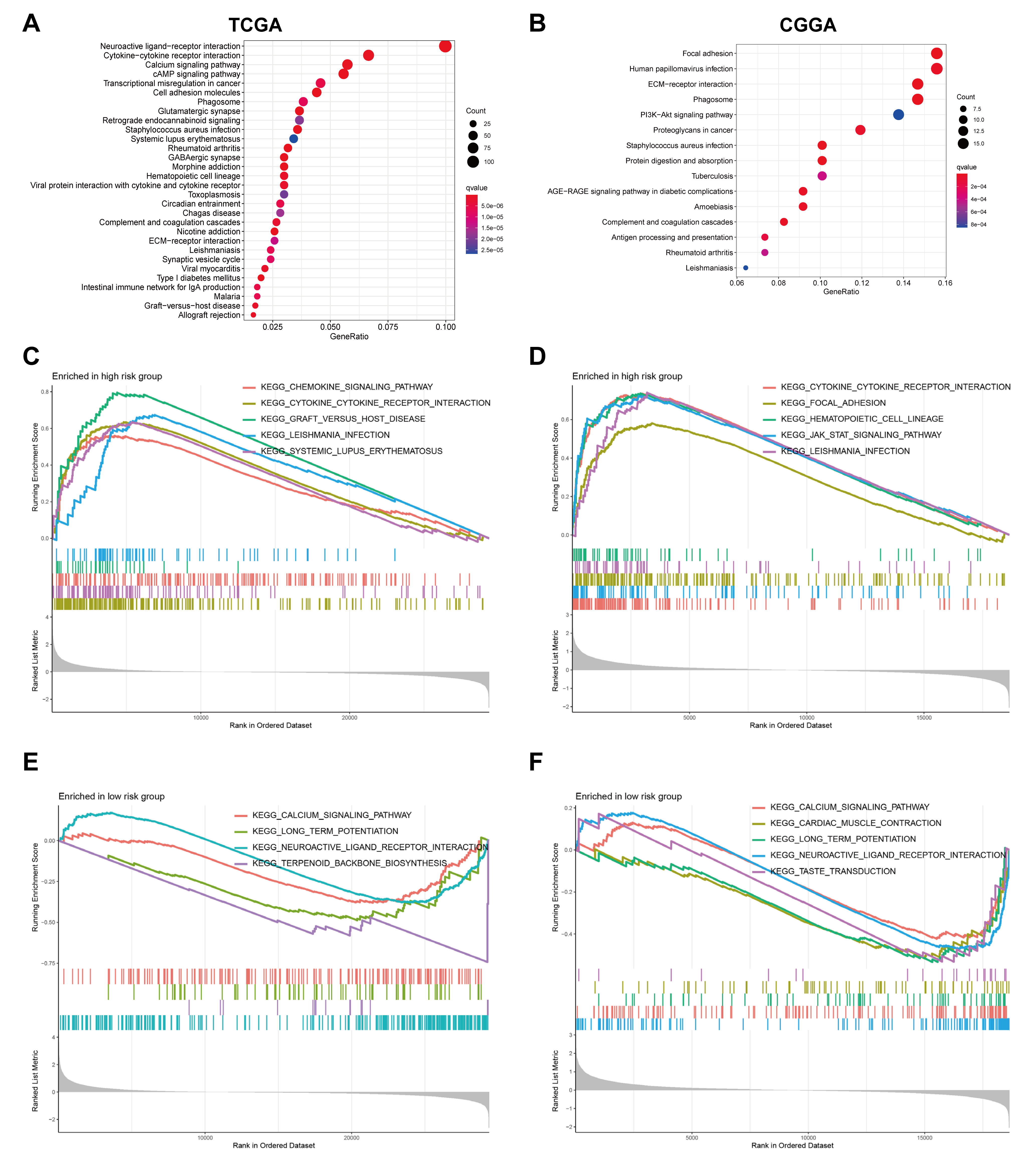

Supplement: Supplementary file 3 [file Image4.TIF]

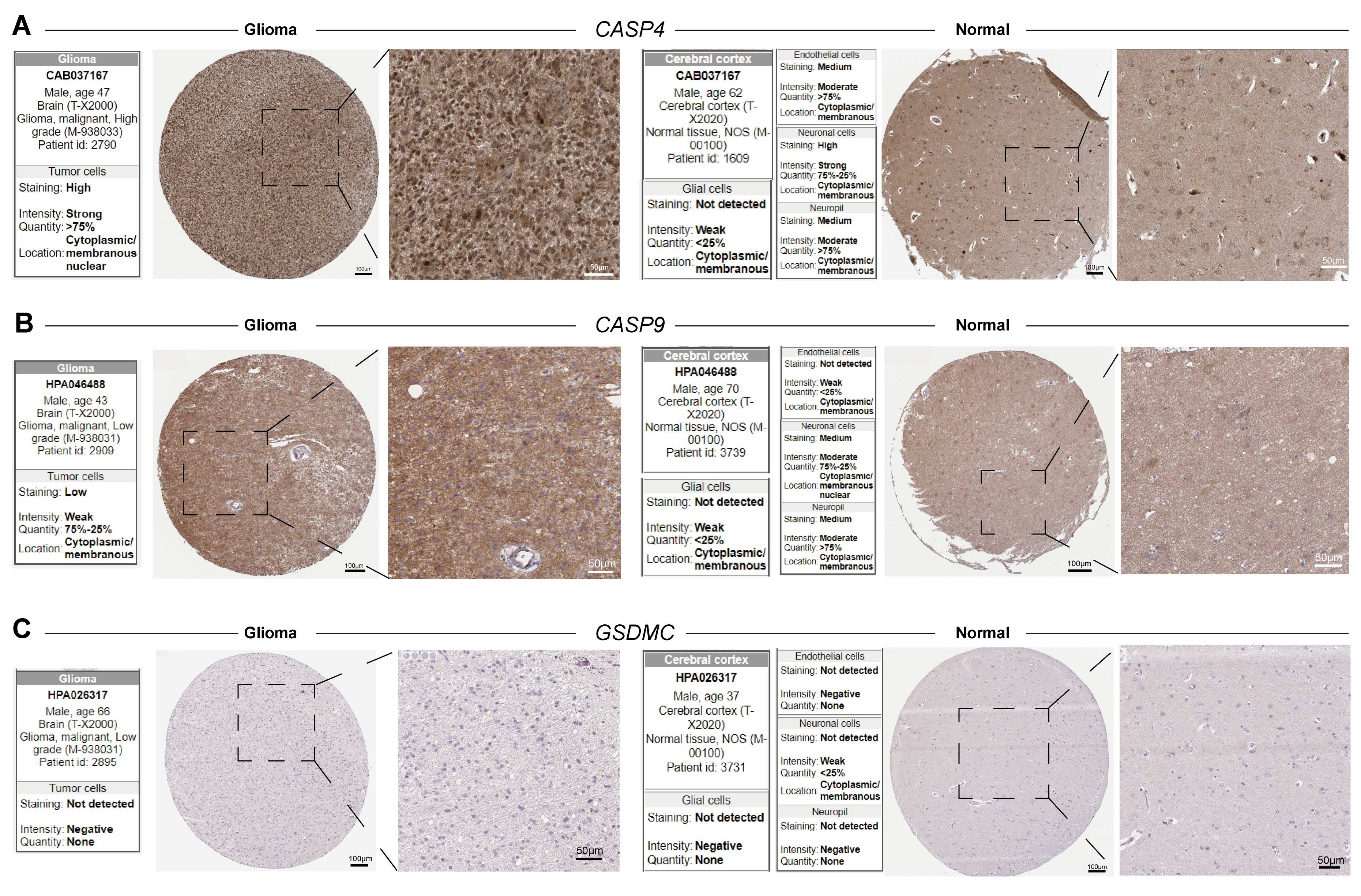

Supplement: Supplementary file 4 [file Image2.TIF]

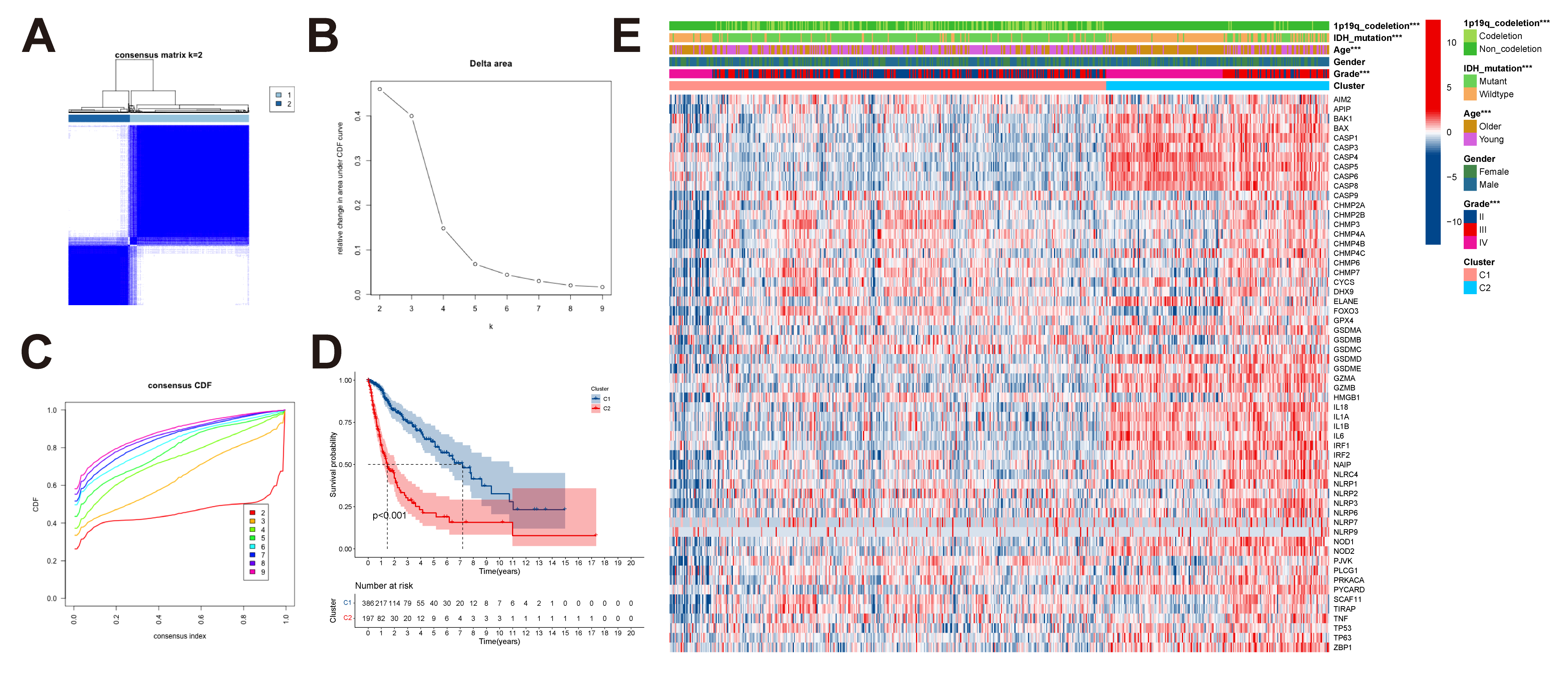

Supplement: Supplementary file 5 [file Image1.TIF]

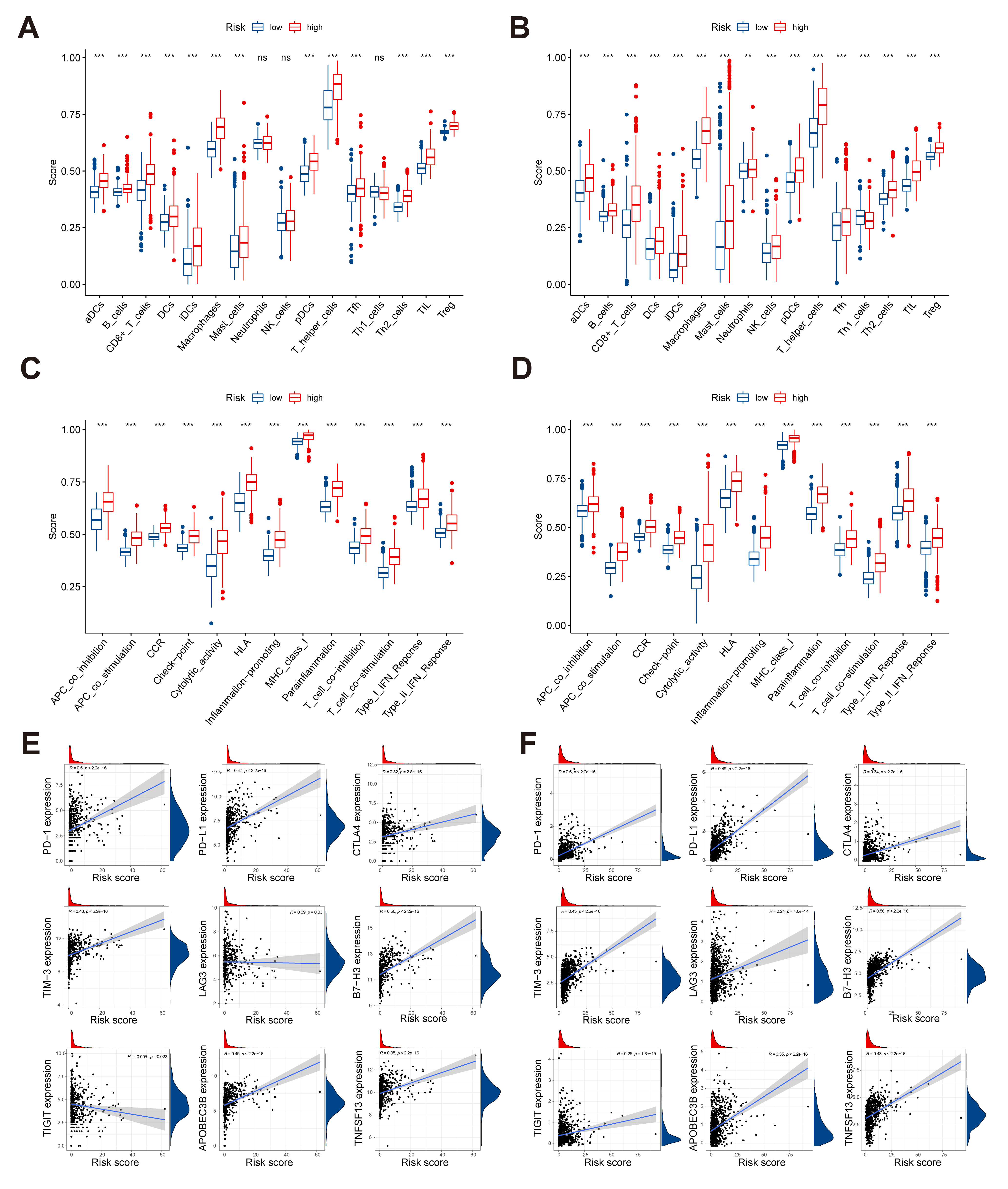

Supplement: Supplementary file 7 [file Image5.TIF]
