# Supplementary material for: Pyroptosis-Related Gene Signature Predicts Prognosis and Indicates Immune Microenvironment Infiltration in Glioma
Source: Front Cell Dev Biol. 2022 Apr 25;10:862493. doi: 10.3389/fcell.2022.862493 (PMC9081442; doi:10.3389/fcell.2022.862493)
Supplement: Supplementary file 6 [file Table3.DOCX]

**Supplementary Table S2. The Primers sequence used in this study**

| **Name** | **Forward-primer** | **Reverse-primer** |
| --- | --- | --- |
| CASP4 | 5’- AGAGAAGCAACGTATGGCAGGA-3’ | 5’- CACCTCTGCAGGCCTGGACAATGATGAC-3’ |
| CASP9 | 5’- CTTCGTTTCTGCGAACTAACAGG-3’ | 5’- GCACCACTGGGGTAAGGTTT-3’ |
| GSDMC | 5’- TCCATGTTGGAACGCATTAGC-3’ | 5’- CAAACTGACGTAATTTGGTGGC-3’ |
| IL1A | 5’- TGGTAGTAGCAACCAACGGGA-3’ | 5’- ACTTTGATTGAGGGCGTCATTC-3’ |
| GAPDH | 5’- GGAGCGAGATCCCTCCAAAAT-3’ | 5’- GGCTGTTGTCATACTTCTCATGG-3’ |
